# The Effectiveness of Emotionally Focused Therapy on Enhancing Marital Adjustment and Quality of Life among Infertile Couples with Marital Conflicts

**DOI:** 10.22074/ijfs.2015.4245

**Published:** 2015-07-27

**Authors:** Maryam Najafi, Ali Akbar Soleimani, Khodabakhsh Ahmadi, Nasirudin Javidi, Elnaz Hoseini Kamkar

**Affiliations:** 1 Department of Psychology, University of Science and Culture, Tehran, Iran; 2 Behavioral Sciences Research Center, Baqiyatallah University of Medical Sciences, Tehran, Iran; 3 Department of Psychology, Family Therapy, Behavioral Sciences Research Center, Baqiyatallah University of Medical Sciences, Tehran, Iran

**Keywords:** Emotion, Therapy, Adjustment, Infertile, Quality of Life

## Abstract

**Background:**

The purpose of this study is to investigate the efficacy of emotionally fo- cused therapy (EFT-C) on promoting marital adjustment of infertile couples with marital conflicts by improving quality of life.

**Materials and Methods:**

This is a semi-experimental study with a pre- and post–test design in which 30 infertile couples (60 individuals) were chosen by purposive sampling. Couples were randomly divided into two groups, sample and control, of 15 couples each. Next, couples in the sample population answered questionnaires for marital adjustment, sexual satisfaction and quality of life after which they received 10 sessions of EFT-C.

**Results:**

Pre- and post-tests showed that EFT-C had a significant effect on marital adjust- ment and quality of life.

**Conclusion:**

According to the results, EFT-C had a significant, positive effect on en- hancement of marital adjustment. Life quality of infertile couples significantly increased via application of EFT-C. This approach improved the physical, psychological and social relationships of infertile couples and enhanced their social environment.

## Introduction

Despite the alteration of standpoints on sexual behavior in recent centuries, fertility remains of crucial importance and children play an important role in cementing a marital relationship. Fertility, as one of the major reasons for marriage, actually results from human nature and introduces the concept of eternal life (1, 2). Another concept, sterility, as the opposite of fertility is defined as the inability to bear offspring after a year of regular sexual activity without contraception (1-3). This inability is considered a failure and leads to the feeling of imperfection in sexual identity. Sterility often causes the person to feel a loss of control over one’s life, doubting one’s manhood/womanhood and generally damages self-confidence and health (4). Highcosts of treatment, constant anxiety about treatment outcomes, exhaustion from visiting various clinics, societal repercussions, confronting questions about a childless marriage, potential distress during the treatment and fear for missing the spouse or destruction of the family are among the factors which result in multiple psychological complications. These complications include frustration, personal conflict, disappointment, sharp decline in self-esteem, isolation, identity crisis and loss of marital adjustment (5). Marital adjustment is a process commonly composed of: i. Marital satisfaction, ii. Dyadic cohesion, iii. Consensus on matters of importance to marital functioning, iv. Showing affection and warmth toward the spouse and v. Sharing intimacies (6, 7). Couples who are well-adjusted gain great satisfaction out of the marital relationship and think well of the spouse’s habits. They enjoy communicating with family and friends, and ask their help with problems. These couples derive immense sexual pleasure as well (8).

Sterility has physical, financial, emotional and psychological impacts on a person (9). Therefore it seriously undermines self-confidence and results in a sexual identity crisis which results in a decline in life quality (10). Life quality has a wide range of definitions. Some believe that it is the ability of an individual to manage life from his/her standpoint, as such, fertility status and its factors can put social and psychological pressures on the person. It lowers sexual pleasure and life satisfaction, meaning a decline in quality (11). Donald considers quality of life as a descriptive term. Quality of life is a perception that an individual holds of their state of health which has a feeling of contentment within, and is accompanied by happiness and joy (12).

Downey and Mckinney (13) declared that women who sought treatment for fertility problems suffered from greater depression, anxiety and stress, along with decreased dyadic cohesion. Most researchers reported increased marital quarrels among infertile couples, which in some cases led to separation (14). Decreasing familial disputes and striving to gratify the partner’s desires could alleviate these problems (15).

In this regard, a host of researches have confirmed efficacy of couples therapy for decreasing marital conflicts (13). As emotions play a central role in infertile couples’ relationships, the emotionally focused approach is employed as short-term structured counseling of 9-20 sessions. This technique is used because it is a branch of couples therapy and because it takes advantage of emotions to develop the process. This method addresses communication disorders, marital discord, and persuades people to express their emotions and talk over them. From the standpoint of couples therapy, marital distress is mostly caused by negative emotions and attachment injuries (16).

A study on 120 infertile couples in East India suggested that infertile men had problems with their character and social interactions. On the other hand, women displayed symptoms of depression. The study indicated that infertility spoiled gender concept, life quality, marital adjustment, and sexual relationships of the couples (17). Poor quality of marital relationship was followed by a number of social and familial troubles. Sterility along with other differences and problems between couples challenged the mind and social welfare of spouses. In general, a low quality within the marital relationship could be problematic when couples struggled with infertility (18, 19).

As a result, attention to psychological needs of infertile couples is essential for successful infertility treatment. Each partner requires support and empathy while undergoing treatment (20). There are a variety of practices to cope with psychological reactions of infertility, among which is emotionally focused therapy for couples (EFT-C) which merges three approaches of systematic, humanistic (empiricism) and attachment theory. This therapy was founded by Johnson and Greenberg in the early 1980s. Given the major role of emotions in attachment theory, EFT-C emphasizes emotions and employs them to organize interaction patterns (16). Hence, EFT-C concentrates on the emotional relationship of couples as a basis to tackle their problems. EFT-C has a process of 9 steps as follows (21):

Step 1: Evaluation, making contact, and then recognition of tensions between couples from the standpoint of attachment.
Step 2: Identification of the cycle of negative interactions that sustain anxiety and bring about insecure attachment.
Step 3: Discerning the underlying feeling or emotion not yet expressed in couples’ interactions that is being concealed.
Step 4: Reframing the problems resulting from the cycle of negative interactions, unmet urges, needs and emotions in order to explore the cycle.
Step 5: Having access to fears and needs of attachment.
Step 6: Promotion of acceptance by the other spouse.
Step 7: Smoothing the way for expression of needs and wants, and restructuring new models of interaction on the basis of perceptions and knowledge obtained from the process.
Step 8: Providing new solutions for old challenges.
Step 9: Strengthening new positions and patterns of behavior (16).

Thus, showing emotions and attachment needs along with sincere fulfillment by the partner constitute the process of EFT-C and are necessary for change (22, 23).

In light of the points previously mentioned, the current study intends to meet the necessity for enhancing marital adjustment of infertile couples - people who suffer from poor life quality and are locked in marital disputes. In addition, the results can serve as a practical map or a manual for counselors, psychotherapists and family therapists to raise their clients’ self-esteem and show mismatch in communication methods between individuals.

## Materials and Methods

This semi-experimental method with a pre- and post-test design was conducted on a sample group of 15 couples and a control group of the same number. Initially, demographic characteristics of the subjects were collected. Next, they were tested prior to conducting the independent variable (EFTC). According to Johnson’s plan (24), couples in the sample group underwent 10 EFT-C sessions of 120 minutes duration conducted twice per week. By the end of the term, subjects were again tested. To meet ethical standards, a compact course of 4 weeks was offered to volunteers from the control group.

The sample population comprised couples married for 10 years who attended infertility clinics in pursuit of treatment during 2013. According to purposive sampling, 30 couples (60 individuals) were selected in terms of poor marital adjustment and low sexual satisfaction. Couples were then divided into two groups of 15 couples (15 men and 15 women); one as control group and another as sample population.

Infertile couples attended fertility clinics. The data-gathering tools were demographic characteristics and World Health Organization (WHO) quality of life questionnaires.

Data were analyzed using SPSS software (version 18) by application of the methods of mean calculation plus minimum and maximum standard deviation from descriptive statistics and analysis of covariance from inferential statistics (alpha 0.81). In order to use analysis of covariance, first the equality of variances was noted. Hence, the hypothesis was examined by the Levin test. Table 1 points out the treatment protocol used in this study, this protocol is emotionally-focused therapeutic approach, which have been provided to the couple during 10 sessions.

### Ethical consideration

In order to observe ethical considerations, a few tutorial sessions were held over four weeks for the control group that did not receive EFT-C.

### Research tools

#### Questionnaire of Demographic Characteristics:

This questionnaire comprised parameters of age, gender, education level, occupation, income level, cause of infertility, duration of infertility, duration of marriage, number of surgeries, date of last surgery, history of attending psychological or counseling sessions, history of any chronic physical or psychological disorders.

#### Spanier’s Dayadic Adjustment Scale:

This scale (25) consists of 32 questions based on a Likert approach of responding which measures the total score of marital adjustment within a range of 0 to 15. People who score 101 or less, according to Spanier, are supposed to be maladjusted and those with higher scores are considered well-adjusted. In a study by Hassan shahi (26), well-adjusted couples had an average score of 114.7 ± 17.8 whereas the average score of maladjusted couples was 70.7 ± 23.8. Spanier grouped the data into four subscales of marital satisfaction, dyadic consensus, dyadic cohesion, and affectional expression with evaluated validity of 0.94, 0.90, 0.81 and 0.73. The entire scale had a validity of 0.96. Reliability was 0.86 according to Pearson’s correlation coefficients between Spanier’s scale and the Locke-Wallace Marital Adjustment Scale. Hassan shahi (26) evaluated the validity of Spanier’s scale in Iran by calculating the cohesion between the Locke-Wallace Marital Adjustment Scale and Spanier’s scale (25).

**Table 1:** Johnson’s Protocol of emotionally focused therapy (EFT-C) for infertile couples


Step	Purpose	Session	To do

1	Identification	1	Collect general information about the couple; introduce the therapist to the partners, investigate grounds and expectations of participation, define the method of EFT-C in addition to concepts of infertility, conflict, marital adjustment, sexual satisfaction, and life quality, ask the couple for their opinion on the method and concepts; identify negative cycles, assess coupleâ€™s way of dealing with issues, discover attachment blocks as well as personal and interpersonal tensions, evaluate status of marital relationship, sexual satisfaction and quality of life.Task: Pay attention to positive and negative emotions, i.e., joy, happiness, anger, hate, sad-ness, jealousy, anxiety, etc.
		2	Appoint a separate session for each partner to discover significant events and information that is not feasible to discuss in the presence of the other, such as commitment to marriage, extramarital relationship, exporter attachment trauma, assess the fear of revelation.Task: Pay attention to your partner’s cycle of interaction.
2	Change	3	Ascertain interaction patterns and ease acceptance of the experienced emotion, discern every partner's fears of insecure attachment, help each partner with openness and self-disclosure, continue the therapy.Task: Discern pure emotions, thoughts, and sentiment.
		4	Restructure the bond through clarification of key emotional reactions, widen the emotional experience of each spouse to create new ways of interaction, partners should accept new patterns of behavior.Task: Express pure emotions and sentiments.
		5	Task: Deepen the relationship by recognizing recently developed needs of attachment; improve personal health and relationship status, express pure emotions and sentiments.
		6	Establish a safe therapeutic alliance, develop new ways of interaction, promote acceptance of the other, discover deep-seated fears and express needs and wants.
		7	Restructure the emotional experiences of the couple, clear the needs and wants of each partner.Task: Underline strengths and weakness.
3	Stabilization	8	Support couple in finding new solutions to past problems, change problematic manners of behavior, facilitate steps the couple can take to invest in their responsive and accessible positions, sync the inner feelings and concepts to the relationship, encourage in positive reaction.Task: Find new solutions to past problems.
		9	Take advantage of therapeutic achievements within daily life to consolidate intimacy, continue with the therapy and its direction, create secure attachment, discern and support constructive patterns of interaction, help the couple shape a story about their future together.Task: Practice the techniques in daily life.
		10	Ease the end of the treatment, Maintain therapeutic changes, draw a comparison between the past and present cycles of interaction, keep an emotional involvement to the deepest status of relationship.


#### World Health Organization (WHO) Quality of Life-BREF (WHOQOL-BREF):

This scale is comprised of 26 questions in areas of physical health (7), psychological health (6), social relationships (3), and environment (8) along with 2 additional questions about quality of life. WHO developed this widely used questionnaire to assess general domains of health. Every question is rated on a Likert scale from 1 to 5. A higher the score assumes better quality of life. The psychometric quality of the questionnaire has received approval in a large number of countries, including Iran (26-28).

According to reports prepared by the scale-makers of WHO from 15 international centers, Cronbach’s alpha for the quad subscale and the entire questionnaire ranged between 0.73 and 0.89. Rahimi (29) evaluated reliability of the WHOQOL-BREF and determined it to be 0.88 for the entire scale. Cronbach’s alpha of physical health, psychological health, social communication, and quality of life environment were calculated to be 0.88, 0.70, 0.77 and 0.65.

## Results

There were 30 participants (15 couples). Of these, there were 30 (31.7%) individuals with diplomas which was the maximum education level and 10 (11.7%) who had secondary school certificates, as the minimum education level. Duration of marriage in subjects was 10 years. The average age of participants was 33.8 ± 5.03 years. Table 2 shows the pre- and post-test scores on marital adjustment and aspects of quality of life in the control and sample groups and table 3 points out the Kolmogorov-Smirnov Test which has used to determine normality of the data.

As the table indicates there was no significant difference between groups in the subscales of marital adjustment (P>0.05). Therefore both groups were the same at the pre-test. According to table 4, it could be inferred that no significant difference existed between groups in the WHOQOL-BREF at the pre-test stage (P>0.05).

**Table 2:** Average pre-test and post-test scores in control and sample groups


Subscales		Study groups					
		Control		Sample		Total	
		Mean	SD	Mean	SD	Mean	SD

Pre-test	Dyadic satisfaction	22.17	4.32	21.27	4.27	21.72	4.29
	Dyadic cohesion	7.97	2.08	7.37	2.19	7.67	2.14
	Dyadic consensus	24.2	5.93	21.8	6.52	23	6.3
	Affectional expression	4.67	1.3	4.4	1.33	4.53	1.31
	Physical health of the couple	18.87	3.1	19.73	2.03	19.3	2.64
	Psychological health of the couple	15.4	2.4	15.27	1.55	15.33	2.01
	Social Relationships	7.1	1.16	6.87	1.36	6.98	1.26
	Social surrounding	21.97	3.34	22.43	2.1	22.2	2.77
Post-test	Dyadic satisfaction	22.57	4.42	41.03	3.59	31.8	10.13
	Dyadic cohesion	7.63	2.06	20.63	2.2	14.13	6.89
	Dyadic consensus	21.33	7.08	54.1	5.13	37.72	17.62
	Affectional expression	4.3	1.34	11.1	0.99	7.7	3.62
	Physical health of the couple	16.9	3.35	31.67	2.26	24.28	7.97
	Psychological health of the couple	14.23	2.18	28.8	1.92	21.52	7.62
	Social relationships of the couple	7.53	2.73	15.13	3.3	11.33	4.87
	Social surroundings of the couple	20.6	3.91	33.57	4.74	27.08	7.83


Table 5 shows a significant difference between the control and sample groups (P<0.001). According to the results, equality of the variances of the control and sample groups was approved (P>0.05). The result of the covariance analysis for comparison of average scores is shown in table 5. The degree of change as a result of EFT-C was as follows: marital satisfaction (86%), dyadic cohesion (92%), dyadic consensus (90%), affectional expression (87%), physical and psychological health (93%), social relationships (62%) and social surroundings (80%), which represented a significant improvement attributed to EFT-C.

**Table 3:** Kolmogorov-Smirnov test to investigate normality of the data


Scales	Subscales	Statistic	Sample size	P value

Marital adjustment	Dyadic satisfaction	1.298	60	0.069
	Dyadic cohesion	0.909	60	0.38
	Dyadic consensus	0.813	60	0.523
	Affectional expression	1.55	60	0.97
Quality of life	Physical health	1.051	60	0.219
	Psychological health	1.091	60	0.185
	Social relationships	1.097	60	0.186
	Environment	1.016	60	0.253


The data was approved as normal for all variables according to the Kolmogorov-Smirnov test (P>0.05).

**Table 4:** Comparison of the groups in the subscales of marital adjustment and WHOQOL-BREF through the pre-test stage


Subscales	Group	Mean	SD	t *	df **	P value ***

Dyadic satisfaction	Control	22.17	4.32	0.811	58	0.421
	Sample	21.27	4.27			
Dyadic cohesion	Control	7.97	2.08	1.089	58	0.281
	Sample	7.37	2.19			
Dyadic consensus	Control	24.2	5.93	1.491	58	0.141
	Sample	21.8	6.52			
Affectional expression	Control	4.67	1.3	0.787	58	0.434
	Sample	4.4	1.33			
Physical	Control	18.86	3.1	-1.279	58	0.206
	Sample	19.73	2.03			
Psychological	Control	15.4	2.4	0.255	58	0.799
	Sample	15.26	1.55			
Social	Control	7.1	1.15	0.717	58	0.476
	Sample	6.86	1.35			
Environment	Control	21.96	33.3	-0.649	58	0.519
	Sample	22.43	2.09			


WHOQOL-BREF; World Health Organization Quality of Life-BREF, SD; Standard deviation, *; Paired t test, **; Degrees of freedom and ***; Probability of rejecting the null hypothesis.WHOQOL-BREF; World Health Organization Quality of Life-BREF, SD; Standard deviation, *; Paired t test, **; Degrees of freedom and ***; Probability of rejecting the null hypothesis.

**Table 5:** ANCOVA of marital adjustment and WHOQOL-BREF in couples


Aspects	Freedom Pretest	Mean square		F Value		P value		Effect size		Statistical power	
		Pretest	Group membership	Pretest	Group membership	Pretest	Group membership	Pretest	Group membership	Pretest	Group membership

Dyadic satisfaction	1	141.8	5238.84	10.12	373.95	0.002	0.001	0.15	0.86	0.87	1
Dyadic cohesion	1	56.08	61.47	15.38	16.86	0.001	0.001	0.21	0.92	0.97	1
Dyadic consensus	1	417.16	16503.14	13.2	522.54	0.001	0.001	0.188	0.902	0.947	1
Affectional expression	1	42.28	3060.73	5.59	404.7	0.021	0.001	0.089	0.88	0.64	1
Psychological health	1	33.26	3201.08	8.99	865.14	0.004	0.001	0.14	0.94	0.84	1
Social relationships	1	9.083	875.42	0.99	95.62	0.32	0.001	0.017	0.62	0.16	1
Social surrounding	1	116.7	2602.69	10.52	234.6	0.002	0.001	0.16	0.807	0.89	1


ANCOVA; Analysis of convariance and WHOQOL-BREF; World Health Organization Quality of Life-BREF.

## Discussion

According to the results, EFT-C had a significant positive effect on marital adjustment. There was improvement in the dyadic satisfaction, dyadic cohesion, dyadic consensus, affectional expression, dimensions of life quality, physical and psychological health, social relationships, and social surroundings subscales. The difference was observed between the control and sample groups as well as between the pre- and post-test results. Findings of this study were consistent with previous studies. Aarts et al. (30) through their research indicated that scores of anxiety, depression, and poor life quality in fertility clinics were related to each other. Consideration of these factors could create positive experiences. They concluded that EFT-C could improve an infertile couple’s quality of life and decrease the level of anxiety and depression. This influence has been attributed to the power of emotions over marital relationships. Emotions play a key role in an infertile couple’s relationship which deserves attention. It is recommended to apply this approach for 9 to 20 structured sessions, as it is both a branch of couples therapy and focuses on emotions. EFT-C addresses communicative disorders and maladjustment and encourages people to speak about their emotions. From the standpoint of EFT-C, marital distress originates from negative emotions and attachment injuries.

The findings of present study were consistent with results of studies by Soltani et al. (31), Zuccarini et al. (32) and Vizheh et al. (33). According to research by Soltani et al. (31) on the influence of EFT-C on couple intimacy in Shiraz, it was suggested that EFT-C could improve emotional, psychological, sexual, physical, communicative, ethical and mental dimensions of couples. However it had no effect on their spiritual and social dimensions.

The results of present study corresponded with findings of Michelle (34) and Tie and Poulson (35) with respect to dyadic consensus. The results of present study also matched findings of Pinto-Gouveia et al. (36) in terms of mutual affection.

Of note, infertile couples tend to express negative and damaging feelings, remarks, sarcasm and criticism rather than empathy while their spouse is trying to deal with an issue (infertility). According to Morin-Papunen and Koivunen (37), marital satisfaction of infertile couples is significantly lower than fertile couples with regards to mutual affection. Onat and Beji (38), in an investigation into the marital life and relationship of infertile couples, have declared that the stress coming from the inability to conceive negatively influenced the couple’s relationship. EFT-C could significantly improve the sexual relationship of the partners.

With regards to physical health of the couples, our findings were consistent with the results of research by Peterson et al. (39) who stated that counseling could improve physical and psychological health of infertile couples. They asserted that psychological counseling through persuasion of the clients to continue with medical treatments significantly improved both their psychological status and physical health.

Our findings were also compatible with the results of Naamen et al. (40) which revealed that social support and understanding played an important role in psychological health of infertile couples, which motivated the couples to continue with infertility treatment and reduced the call for divorce. Javidi et al. (41) indicated that EFT-C had influential effects on family functioning, inasmuch as this protocol took advantage of a systematic approach which refined the inflexible interaction patterns of couples in distress and strengthened their bonds. Soltani et al. (42) concluded that EFT-C was capable of promoting marital adjustment of infertile couples.

Najafi et al. (43) evaluated studies about questionnaires of life quality among infertile couples. Through screening all studies, they found 10 general and 2 specialized inventories. Although no meta-analysis was found, infertility negatively influenced couples’ quality of life. This research indicated that general questionnaires (SF-36, WHO-QoL, and FERTI-QoL) were mostly used for evaluation of infertile couples quality of life.

Ramezanzadeh et al. (44) investigated the emotional adjustment of infertile couples. They concluded that people unable to conceive suffered from psychiatric disorders (particularly stress and depression) which led to emotional maladjustment.

## Conclusion

EFT trains couples to give stronger support to each other, corrects their patterns of behavior and raises their accessibility accompanied by responsiveness to the partner’s needs in order to achieve an optimal sexual relationship.

The present study showed that EFT-C significantly increased satisfaction, cohesion, consensus and affection expression of the partners. The life quality of infertile couples remarkably grew which was attributed to EFT-C. This method improved the social relationships of infertile couples and improved their physical and psychological health. The findings have opened a window for family therapists to conduct more practical and clear counseling in order to improve their client’s selfworth and assist with self-disclosure, as well as revision of wrong communicative patterns which can lead to a decline in marital disputes and increase in adjustment. In addition, the results have shown that EFT-C has a significant effect on enhancement of sexual satisfaction in infertile couples by increasing physical sexual satisfaction as well as emotional sexual satisfaction.

We recommend that similar research be conducted with different populations (in addition to infertile couples) that have diverse levels of education. In addition, follow-ups should be conducted at later months in order to compare the results.

Constraints of the study included the enrollment of couples that had a minimum educational level of a diploma. Hence, to generalize the results of the present study to illiterate individuals, measures of prudence should be taken. The study was performed in one province. Generalization of the findings to other statistical areas should be made with caution.
